# Small molecular floribundiquinone B derived from medicinal plants inhibits acetylcholinesterase activity

**DOI:** 10.18632/oncotarget.19169

**Published:** 2017-07-11

**Authors:** Bing Niu, Mengying Zhang, Pu Du, Li Jiang, Rui Qin, Qiang Su, Fuxue Chen, Dongshu Du, Yilai Shu, Kuo-Chen Chou

**Affiliations:** ^1^ Shanghai Key Laboratory of Bio-Energy Crops, College of Life Science, Shanghai University, Shanghai, 200444, China; ^2^ Center for Informational Biology, University of Electronic Science and Technology of China, Chengdu, 610054, China; ^3^ Gordon Life Science Institute, Boston, MA 02478, USA; ^4^ Department of Neurology, The First People's Hospital of Foshan, Foshan, 528000, China; ^5^ Department of Otolaryngology–Head and Neck Surgery, Eye and Ear, Nose, Throat, Hospital, Shanghai Medical College, Fudan University, Shanghai, 200031, China; ^6^ Department of Gynecology, Affiliated Minzu Hospital of Guangxi Medical University, Minzu Hospital of Guangxi Zhuang Autonomous Region, Nanning, 530001, China; ^7^ Department of Life Science, Heze University, Heze, Shandong, 274500, China

**Keywords:** Alzheimer’s disease (AD), floribundiquinone B (FB), plant inhibitor, acetylcholinesterase (AChE), fluorescence quenching

## Abstract

Being a neurodegenerative disorder, Alzheimer's disease (AD) is the one of the most terrible diseases. And acetylcholinesterase (AChE) is considered as an important target for treating AD. Acetylcholinesterase inhibitors (AChEI) are considered to be one of the effective drugs for the treatment of AD. The aim of this study is to find a novel potential AChEI as a drug for the treatment of AD. In this study, instead of using the synthetic compounds, we used those extracted from plants to investigate the interaction between floribundiquinone B (FB) and AChE by means of both the experimental approach such as fluorescence spectra, ultraviolet-visible (UV-vis) absorption spectrometry, circular dichroism (CD) and the theoretical approaches such as molecular docking. The findings reported here have provided many useful clues and hints for designing more effective and less toxic drugs against Alzheimer's disease.

## INTRODUCTION

With the aging of the population, the elderly related diseases become increasingly prominent. Alzheimer's disease (AD) [1], a degenerative disorder of the central nervous system with the loss of memory and dysfunctions of language and behavior, is the most common form of senile dementia. It is predicted that one in eighty-five people will be living with the disease by 2050 [1]. The incidence of AD in senectitude shows the elevated trend year by year. Therefore, the identification and development of novel and revolutionary therapeutic strategies and drugs are definitely needed [2].

The knowledge of protein three-dimensional structures is vitally important for understanding crucial molecular mechanisms [3–7] for rational drug design [8, 9] and computational modeling [10]. Although many studies had been conducted during the last two decades or so (see, e.g., [11–21]), the pathogenesis of AD is still elusive and so far there is no effective prevention and treatment. Nevertheless, there are many hypotheses about its pathogenesis, such as free-radical injury hypothesis [22], amyloid peptide hypothesis [11, 23], cholinergic hypothesis [24] and Tau hyper-phosphorylation hypothesis [25]. Cholinergic hypothesis [24], the oldest one trying to explain the cause of the disease, is one of the most important theory for currently available drug therapies. It supposes that the change of cholinergic system is closely related to the degree of cognitive impairment. Therefore, acetylcholinesterase (AChE) has been considered as an effective target for the treatment of AD [24], and hence acetylcholinesterase inhibitor (AChEI) was also regarded as an important agent for AD therapy.

Several drugs for treating AD in clinical therapy have been investigated including Tacrine [26], Donepezil [27] and Rivastigmine [28]. Although good effects have been achieved by using these drugs, there are some shortcomings, such as: the high cost of synthesis, limited selectivity, weak performance, toxic side effects, et al. [29]. In recent years, with the development of the separation, purification and analysis technology, the research of natural active ingredients for preventing and treating Alzheimer's Disease has also achieved new progresses [30–32]. Several effective AChEIs such as Huperzine-A [33], Galanthamine [34] and Physostigmine [35] derived from medicinal plants showed that they possessed the advantages of higher inhibitory activity and insignificant toxicity and side effects.

**Figure 1:**
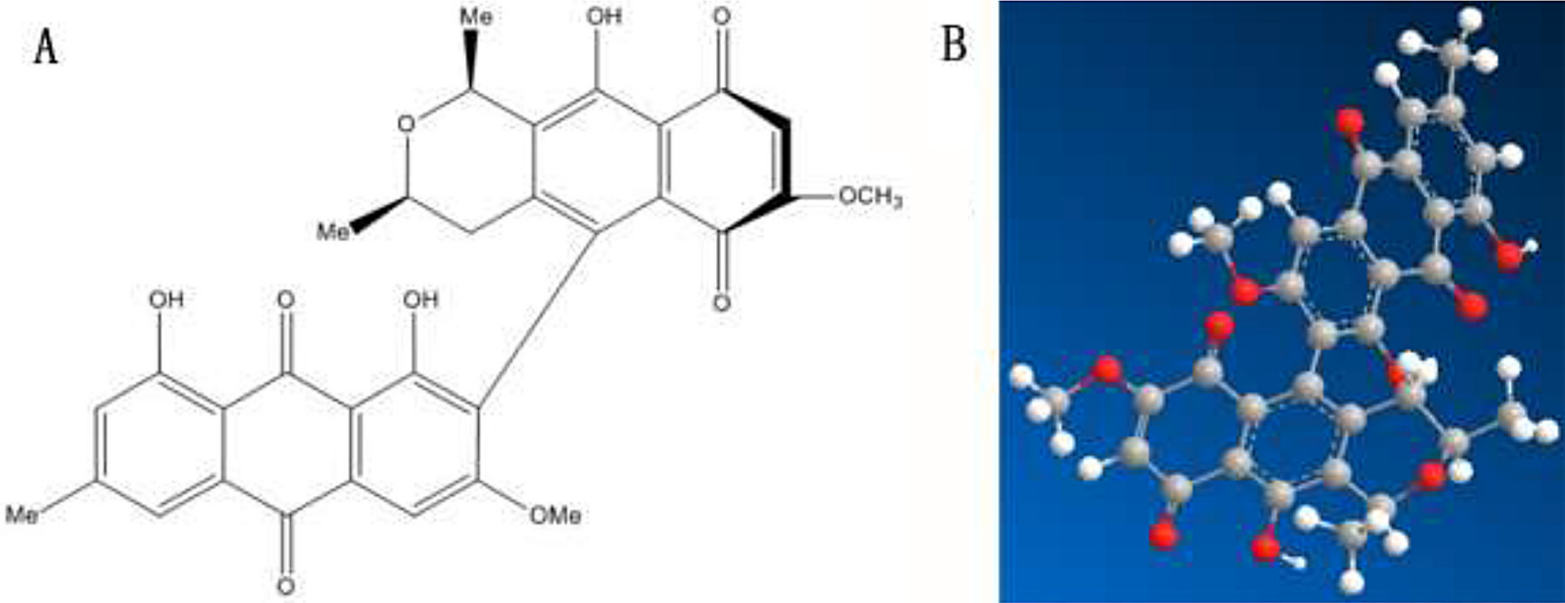
Molecular structures of FB

In 2008, Wei et al. [36] discovered four new compounds from the roots of Berchemia, and named them Floribundiquinone A (FA), Floribundiquinone B (FB), Floribundiquinone C (FC), and Floribundiquinone D (FD), respectively. Since FB is easier to acquire, in this study we are mainly focused on the inhibition of FB (Figure 1) for AChE. Both the experimental method (such as fluorescence spectra [37, 38], ultraviolet-visible (UV-vis) absorption spectrometry [39], circular dichroism (CD) spectrum [40]) and theoretical method (such as molecular docking [41–55]) were applied to study and analyze the interaction of FB-AChE and the binding site. Furthermore, the Topomer CoMFA and Ellman [56] were also used to analyze the biological activity of FB.

## RESULTS AND DISCUSSION

### The interactions between FB and AChE

#### Fluorescence quenching of AChE

**Figure 2:**
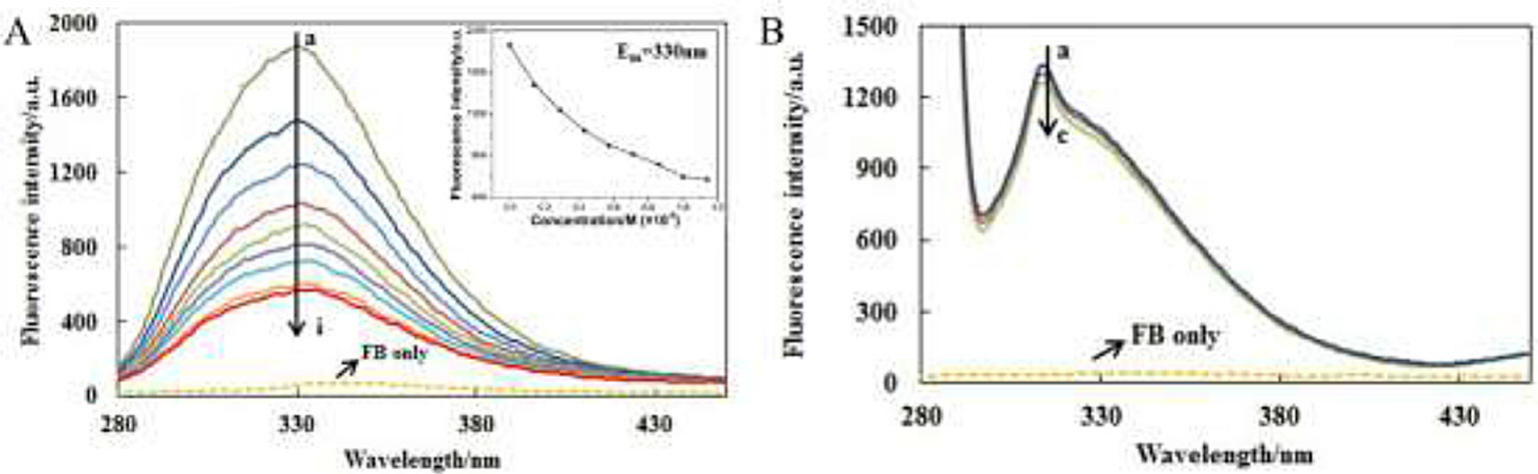
The fluorescence emission spectra of AChE-FB with excitation wavelength of 230 nm (**A**) and 280 nm (**B**). C_AChE_ = 5 × 10^−8^M,C_FB_,a→i: 0, 0.14, 0.29, 0.43, 0.57, 0.71, 0.86, 1 and 1.14 × 10^−6^M; C_FB_,a→c: 0, 0.14, and 0.29 × 10^−6^M; T = 303 K.

The fluorescence emission spectra of AChE were recorded at different concentrations of FB with the excitation wavelength of 230nm (Figure 2A) and 280 nm (Figure 2B) at three temperatures (298 K, 303 K and 308 K) and one of them (303 K) is shown in Figure 2. The fluorescence spectra of the polypeptide backbone structure and excited tyrosine and tryptophan are shown in Figure 2A and Figure 2B, respectively. From Figure 2A (230 nm), it can be seen that AChE is with a strong fluorescence emission peak, while FB with very weak intrinsic fluorescence under the same experimental conditions. Besides, the fluorescence intensity of AChE decreases gradually along with the increase of FB concentration. Hence, it can be concluded that the interaction between AChE and FB does exist, and the combination of AChE and FB becomes saturated gradually along with the increase of FB concentration. Under the effect of FB, the maximum emission wavelength of AChE was accompanied by a slight red shift (330 nm→335 nm), proving that FB can induce the microenvironmental change around AChE polypeptide backbone and result in the enhanced of polarity. It can be seen from Figure 2B (280 nm) that there was a strong fluorescence emission peak of AChE as well, and that the intrinsic fluorescence of FB was also very weak under the same experimental conditions. Moreover, the fluorescence intensity of AChE dropped gradually along with the increase of FB concentration, indicating the effect of FB on the tyrosine and tryptophan residues of AChE.

Fluorescence quenching here refers to the quenching between the fluorescent molecules and the solvent molecules. The fluorescence quenching can be classified as static quenching and dynamic quenching. To further confirm the quenching mechanism, we analyzed the fluorescence quenching of AChE at different temperatures (298 K, 303 K and 308 K) with λ_ex_ = 230 nm by means of the following Ster-Volmer equation

$$F0/F=1+K_{q}\tau_{0}[Q]=1+K_{sv}[Q]$$ (1)

where F_*0*_ and F are the fluorescence intensity before and after the quencher, respectively, k_*q*_ is the rate constant in fluorescence quenching process; [Q] is the concentration of quencher; K_*sv*_ is the quenching constant, τ_*0*_ is the life expectancy of a fluorescent molecule (generally the 10^−8^ s) without quencher [57, 58].

The Stern–Volmer curves of the fluorescence quenching of AChE by FB at different temperatures are given in Supplementary Figure 1 of Supporting Information. Shown in Table 1 are the quenching constant *K*_sv_ and correlation coefficient of AChE obtained by the linear equation. It has been observed that *K*_sv_ is reduced along with the increasing of temperature, and that *k*_q_ is 22.87 × 10^14^, 20.97 × 10^14^, 19.11 × 10^14^ L/(mols) at 298 K, 303 K and 308 K, respectively. And they are significantly greater than the maximum quenching rate constants of the biomolecule (2.0 × 10^10^L/(mols)) [59]. All these results have clearly demonstrated that the fluorescence quenching of AChE by FB is static quenching that may cause the reaction between quenching agent and fluorescent material molecules of the ground state.

**Table 1:** The quenching constant K_sv_ and correlation coefficient of AChE

T(K)	*K*_SV_ (× 10^6^M^−1^)	*k_q_* (×10^14^M^−1^S^−1^)	R^a^
298	22.84	22.84	0.997
303	20.97	20.97	0.994
308	19.11	19.11	0.995

R^a^ is the linear correlated coefficient.

### UV-vis absorption spectra of FB and AChE system

UV-vis absorption spectroscopy [60, 61] is a simple and effective way for detecting the protein conformational changes and the complex formation. The quenching mechanism of the drug and AChE may be further verified by the UV-vis absorption spectroscopy method.

From Supplementary Figure 2 of Supporting Information, it can be observed that AChE has a strong absorption within the wavelength range of 200 nm-240 nm, the band that reflects the information of protein backbone. The intensity of the absorption peak of AChE at 230 nm is remarkably reduced along with the increase of FB concentration, and the absorption peak has a slight red shift. There is a weak absorption peak at 280 nm, which is mainly the absorption peak of the conjugated double bonds in tyrosine and tryptophan residues of AChE. These results indicate that the interaction of FB with AChE may change the conformation of AChE and the microenvironment, and hence changing the hydrophobicity. Moreover, it has also confirmed that FB does interact with AChE to form a ground-state complex and that the fluorescence quenching is mainly a static quenching process.

### Calculation of binding constant and binding sites

Static fluorescence quenching means that fluorescent donor molecule and a fluorescent quencher molecule combine to form a ground state complex with certain structure, while non-fluorescent by intermolecular force would lead to the phenomenon of the decrease of fluorescence. The supposed fluorescence quenching of protein is static quenching. The number of binding sites (*n*_b_) can be determined by the following equation

$$\mathrm{lg}[(F0/F))/F]=\mathrm{lg}K+n_{\text{b}}\mathrm{lg}[Q]$$ (2)

The binding affinity of drugs and AChE was determined by the binding constant. The size of the value would reflect the binding strength, and it would have a direct impact on the distribution and elimination of drugs. Thus, it will have an impact on the intensity and duration of drug action as well.

When, Eq.2 can be used to calculate the binding constant (K) between the drug and the protein [62]; i.e.,

$$F_{0}/(F_{0}-F)=1+K^{-1}){\left[Q\right]}^{-1}$$ (3)

Shown in Supplementary Figure 3 of Supporting Information are (A) the corresponding double logarithmic curve and (B) the modified Stern-Volmer curves. It can be seen from Table 2 that the binding sites of FB are approximately equal to 1, implying that the AChE has a single binding site for FB to bind, and the binding constant is reduced by the temperature increase, coinciding with the change of quenching constant.

**Table 2:** The binding sites (n) and the binding constant (K) of the inaction of AChE and FB

T(K)	*K* (× 10^6^M^−1^)	*n*	R^a^
298	22.47	1.02	0.994
303	19.19	1.04	0.998
308	14.72	1.10	0.998

R^a^ is the linear correlated coefficient.

### Fluorescence resonance energy transfer between FB and AChE

According to the Förster theory [63], there is enough overlap between the fluorescence spectra of the donor (AChE) and the UV absorption spectrum of acceptor (FB). When the distance between the donor (AChE) and the acceptor (FB) was less than 7 nm, the non-radiative energy transfer will occur and hence result in fluorescence quenching. The relationships of non-radiative energy transfer efficiency (E), the distance (r) between the donor and the acceptor and the critical energy transfer distance (R_0_) are as follows

$$E=1-F/F_{0}=R_{0}^{6}/(R_{0}^{6}+{\text{r}}^{6})$$ (4)

$$ER_{0}^{6}=8.8\times 10^{-25})(K^{2}\cdot \Phi \cdot n^{-}4\cdot \text{J})$$ (5)

$$EJ=\frac{\sum F_{\lambda }\varepsilon_{\lambda }\lambda^{4}\Delta \lambda }{\sum F_{\lambda }\varepsilon_{\lambda }\lambda^{4}\Delta \lambda }$$ (6)

where F_0_ and F are the fluorescence intensity before and after the quencher reaction; R_0_ [64] is the critical distance when the non-radiative energy transfer efficiency is 50%; K^2^ is the dipole spatial orientation factor and its value is taken as 2/3 for random orientations; n is the refractive index of the medium and its value is taken as 1.336 [65] for diluted solution; Φ is the fluorescence quantum yield of the donor and its value is taken as 0.15 [66]; J is the overlap integral of the donor's fluorescence emission spectrum and the acceptor's absorption spectrum; F_λ_ is the fluorescence intensity of AChE on the wavelength at λ, and ε_λ_ is the molar absorption coefficient of the acceptor upon the wavelength at λ [64].

**Figure 3:**
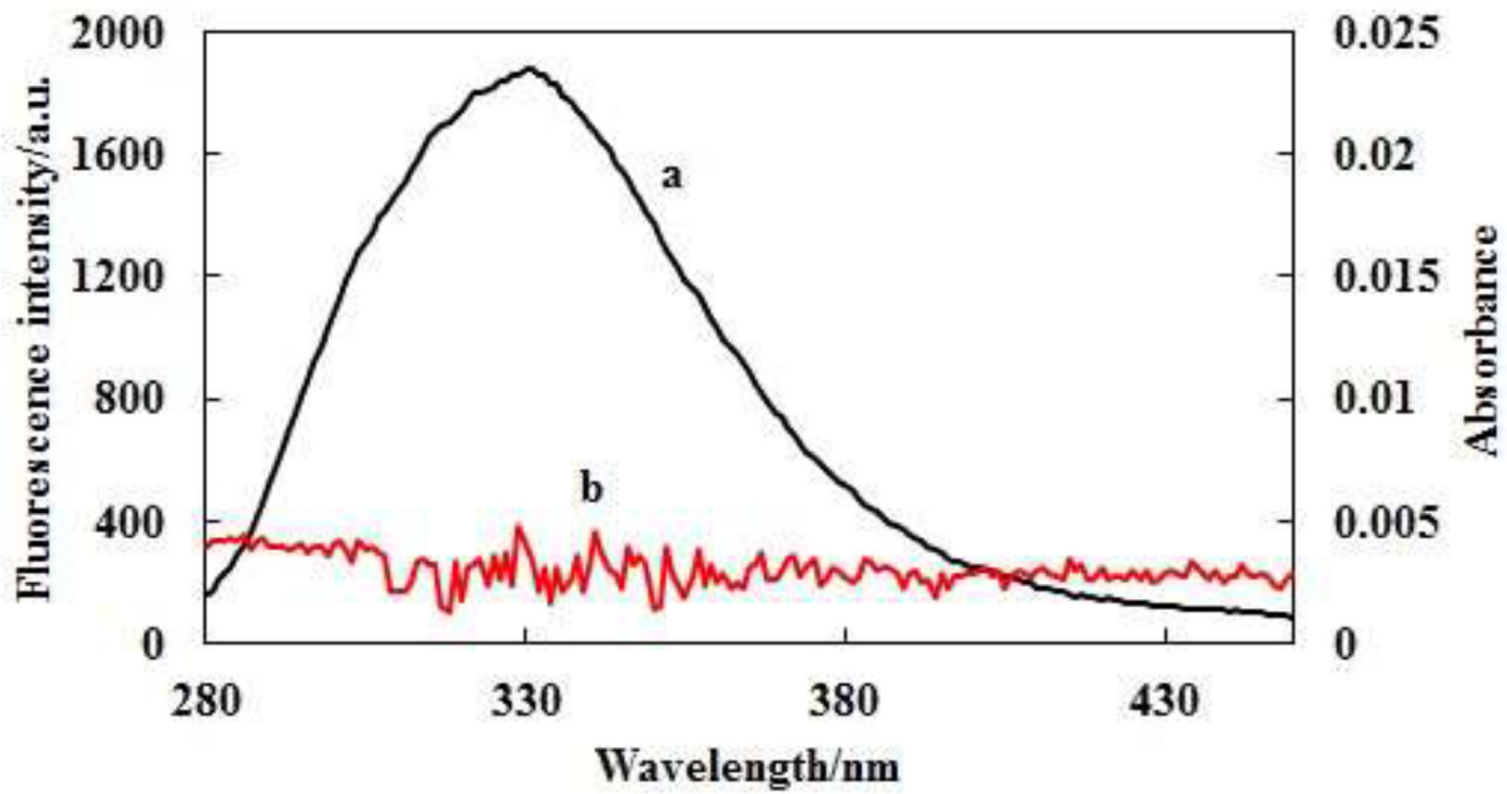
Overlap of the fluorescence spectra of AChE (**A**) and the absorption spectrum of FB (**B**).

Shown in Figure 3 are (a) the overlap of the fluorescence spectra of AChE and (b) the absorption spectrum of FB . Thus, the corresponding parameters (J = 8.3589 × 10^−14^ cm^3^L/mol, R_*0*_ = 3.49nm, E = 0.4961, r = 3.502nm) of FB were obtained according to the Eqs.4–6. The distance between the donor (AChE) and the acceptor (FB) was less than 7nm, and 0.5R_0_ < r < 1.5R_0_, indicating the possibility of energy transfer between FB and AChE was high [60].

It is instructive to point out that similar energy transfer via resonance was also been analyzed in protein/DNA systems [67–72] and used to reveal some marvelous biological functions in biomacromolecules [2, 73].

### Effect of FB on the AChE conformation

**Figure 4:**
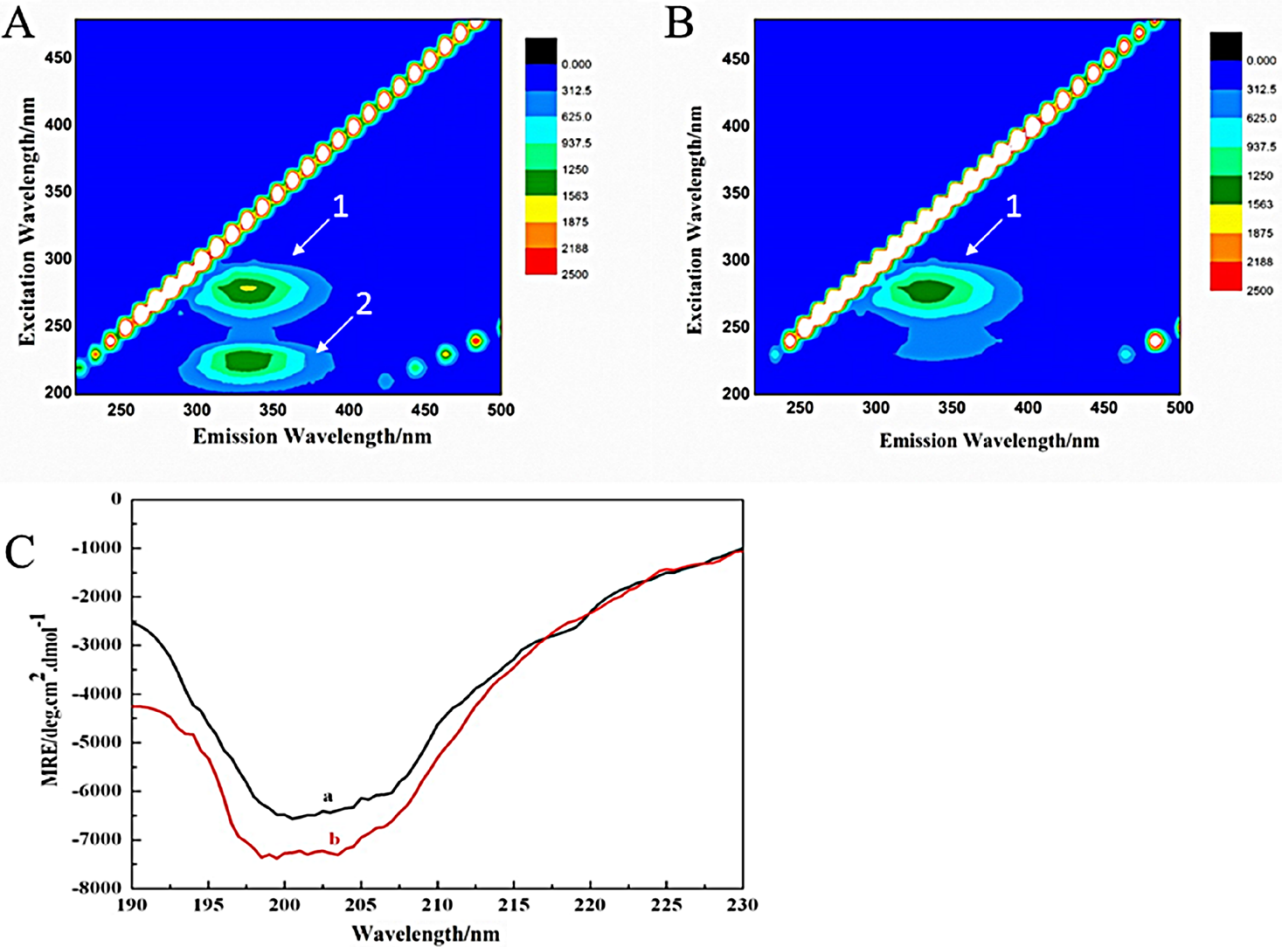
Three-dimensional fluorescence spectra contour maps of AChE (**A**) and the FB-AChE system (**B**), the CD spectra (**C**) of AChE (A) and FB-AChE (B) System.

Aforementioned UV and fluorescence experiments showed that FB did affect the main peptide chain of AChE. To further study the effect of FB on the AChE conformation, the effects of FB on the tertiary structure of AChE were investigated by the 3D (three-dimensional) fluorescence spectroscopy. The fluorescence information of the sample can be fully demonstrated by the 3D fluorescence spectra [36]. The 3D fluorescence spectra and its corresponding contour maps of AChE and FB-AChE systems (molar ratio 2: 1) were shown in Figure 4 (A and B) and Supplementary Figure 4. Peak 1 is the endogenous fluorescence peak of AChE when the excitation wavelength was 280 nm, which mainly reflects the spectral characteristics of tyrosine and tryptophan residues. The fluorescence intensity is slightly and gradually decreased after dropping FB with a red shift at about 2nm, indicating that the FB may change the microenvironment around tyrosine and tryptophan residues of AChE and increase the polarity. Peak 2 is endogenous fluorescence peak of AChE when the excitation wavelength was 230 nm, reflecting the fluorescence spectrum of peptide backbone structure. The fluorescence intensity is greatly and gradually reduced after dropping FB with a red shift at about 4nm, implying that the FB may affect the microenvironment around peptide backbone structure. In other words, the microenvironment hydrophobicity around the peptide chain is reduced and the polarity is increased.

In this study, we also used the CD [40] to investigate the impact of FB on the secondary structure of AChE due to its binding to latter. And the CD spectra of far-UV between 190–230 nm band was selected that is related to the conformation of AChE. As seen in Figure 4C, the peak of AChE in the vicinity of 200 nmis regarded as characteristic negative peaks of random coil. The CD spectra of AChE was changed after dropping FB. The contents of different secondary structures of AChE were quantitatively analyzed by CONTINLL algorithm using CDPro software (Table 3). The contents of α-helix, β-turn and random coil were increased, but the content of β- folded was decreased. The changes in the secondary structures show a full consistency with the above experimental results.

**Table 3:** The content of different secondary structures of AChE obtaining by CONTINLL algorithm

Compounds	*H*(*r*) (%)	*H*(*d*) (%)	*S*(*r*) (%)	*S*(*d*) (%)	Trn (%)	Unrd (%)
AChE	0.3	5.2	23.9	12.8	22.6	35.2
AChE+FB	0.9	5.8	21.4	12.6	23.1	36.2

H(r): Regular α- helical structure; H(d): Irregular α- helical structure; S(r): Regular β-sheet structure; S(d): Irregular β-folded structure; Trn: β- turn structure; Unrd: Random coil structure.

### Analysis with molecular docking

**Figure 5:**
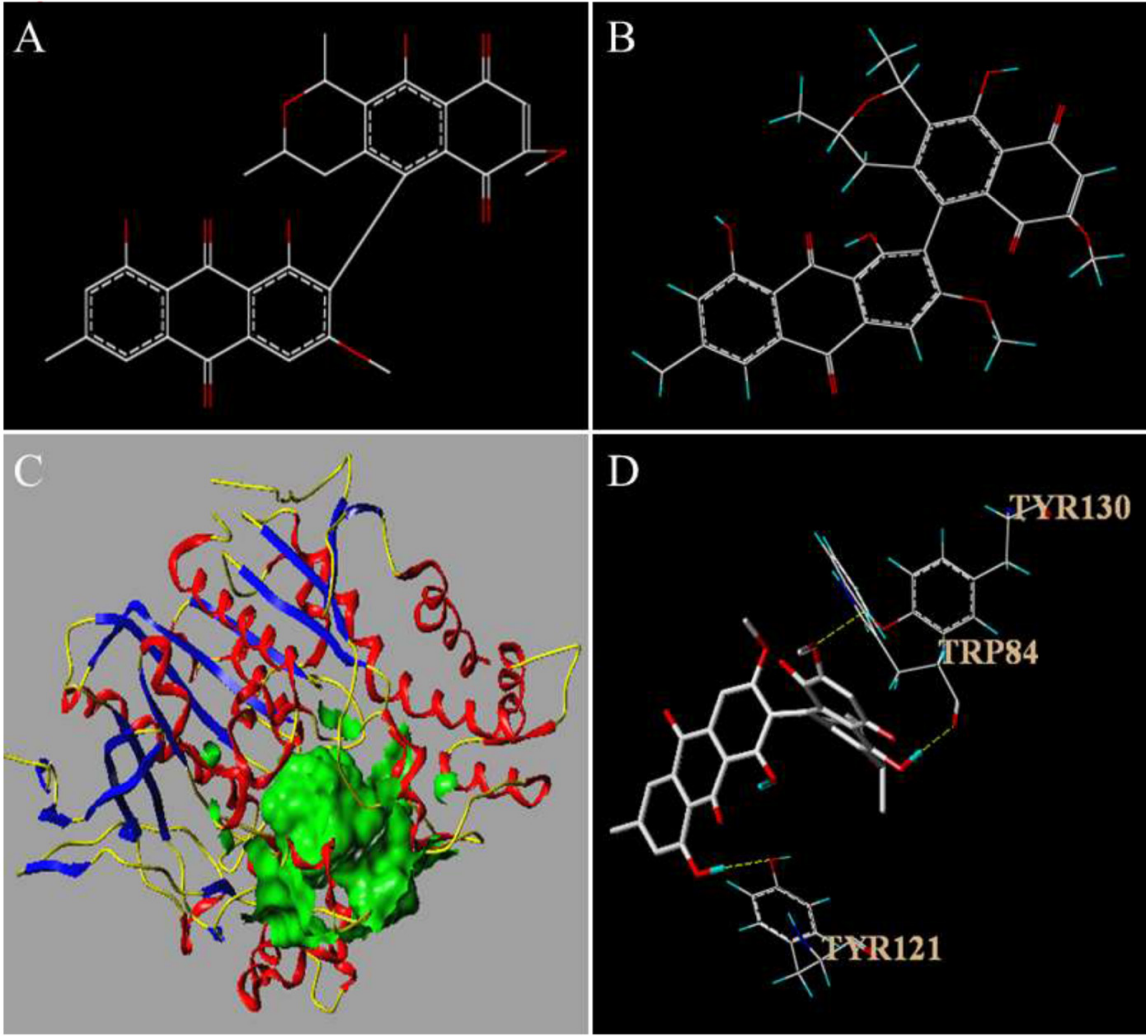
The structure of FB before (**A**) and after (**B**) optimization in SYBYL-X-2.0. The active pocket of AChE (**C**) and the results of the molecular docking (**D**). In Figure 5C, the ligand is shown in green and the secondary structure of protein is shown in yellow, blue and red ribbon. In Figure 5D, hydrogen bonding depicted in yellow dashed lines.

Molecular docking is a useful vehicle for investigating the interaction of a protein receptor with its ligand and revealing their binding mechanism as demonstrated by a series of studies (see, e.g., [16, 41–43, 46–54, 74–77]). In this study, molecular docking of FB with AChE (PDB ID: 1QTI) was studied by using the Tripos molecular modeling package in SYBYL-X-2.0. The results thus obtained are shown in Figure 5, where we can see that the binding of FB to AChE is mainly through hydrogen bonds and that there are three binding sites between the FB and AChE. Also, FB can interact with TYR121; the latter is one of the peripheral anionic sites (PAS) of the AChE [78]. The clinical drugs such as Tacrine, Rivastigmine and Huperzine A also have a strong interaction with the PAS [79, 80] of AChE. TRP84 is the binding site of choline to AChE, and can hydrolyze Ach thereby to reduce the affinity of enzyme-substrate as well as the enzyme's activity. Meanwhile, the score of molecular docking was also obtained. The “TotalScore” was 5.9157. Note that it means that the interaction between small molecule and macromolecule is very strong when the “TotalScore” is more than 4. Accordingly, the interaction between FB and AChE is very strong.

### Analysis of biological activity of FB

#### Predicting and validating the activity of FB

The training dataset was used to build Topomer CoMFA model by fragmenting them into R1 and R2 groups. And the testing dataset was adopted to evaluate the stability and predictive ability of the model. The experimental and predicted pIC_50_ values calculated by the model were compared with each other, and their correlations are shown in Table 4. The success rates by the jackknife [81] or LOO cross-validation on the training dataset and testing dataset were 0.990 and 0.899, respectively. And the MRE of the training dataset and testing dataset were 2.88% and 6.40%, respectively. All these results indicate that the Topomer CoMFA model is a very effective predictor. Accordingly, the predicted result pIC_50_ = 5.95 for FB is quite reliable as well.

**Table 4:** The experimental pIC_50_, predicted pIC_50_, R1, R2 values of TopomerCoMFA model

Compounds	Exp	Pred	R1	R2
	Training dataset			
1	8.97	9.41	1.47	2.17
2	8.59	8.56	1.47	1.32
3	8.67	8.46	0.53	2.17
4	9.06	8.93	0.53	2.63
5	8.39	8.35	0.53	2.05
6	9.49	9.48	−2.09	5.81
7	9.52	9.46	−2.09	5.79
8	5.50	5.01	−2.09	1.34
9	3.25	3.51	−2.09	−0.17
10	2.98	3.01	−2.09	−0.67
11	3.23	3.79	−2.09	0.12
12	3.36	3.86	−2.09	0.19
13	3.44	3.33	−2.09	−0.34
14	5.11	4.98	−0.83	0.06
15	3.04	2.89	−2.09	−0.79
16	3.20	3.35	−2.09	−0.32
17	3.41	3.30	−2.09	−0.37
18	3.82	3.69	−2.09	0.02
19	4.99	5.03	−2.09	1.36
20	5.54	5.44	−2.09	1.76
21	5.11	4.74	−2.09	1.05
22	5.20	4.91	−2.09	1.24
23	5.57	5.60	−2.09	1.92
24	7.39	7.31	1.05	0.49
25	8.29	8.35	0.92	1.66
26	8.28	8.25	0.92	1.56
27	8.25	8.17	0.92	1.49
28	7.77	7.92	0.92	1.23
29	7.05	7.09	0.92	0.40
30	7.29	7.18	0.92	0.49
31	7.58	7.78	0.10	1.91
32	7.68	7.85	0.10	1.99
33	7.91	7.97	−2.09	4.29
34	7.93	7.74	−2.09	4.06
35	8.17	8.13	−2.09	4.46
36	9.49	9.48	−2.09	5.81
37	8.85	9.13	−2.09	5.45
38	7.89	7.78	1.47	0.54
39	6.89	6.77	0.46	0.54
40	6.41	6.54	0.22	0.54
41	5.32	5.27	−0.09	−0.41
42	6.77	6.83	1.47	−0.41
43	7.67	7.82	1.47	0.58
44	9.60	9.14	1.47	1.90
45	7.49	7.74	0.53	1.45
46	7.52	7.57	0.53	1.27
47	8.16	8.19	0.53	1.90
	**Testing dataset**			
48	9.57	9.87	1.47	2.63
49	9.17	9.29	1.47	2.05
50	5.66	4.96	−2.09	1.29
51	5.47	5.52	−2.09	1.84
52	5.63	5.15	−0.67	0.06
53	7.23	8.05	1.05	1.23
54	7.53	7.06	0.10	1.19
55	7.56	7.28	0.10	1.41
56	7.35	7.76	1.45	0.54
57	6.34	6.71	0.40	0.54
58	6.54	6.22	−0.09	0.54
59	5.43	5.59	0.22	−0.41
60	7.42	8.23	0.53	1.93
61	7.45	8.32	0.53	2.02
**FB**	/	5.95	1.21	0.17

**Figure 6:**
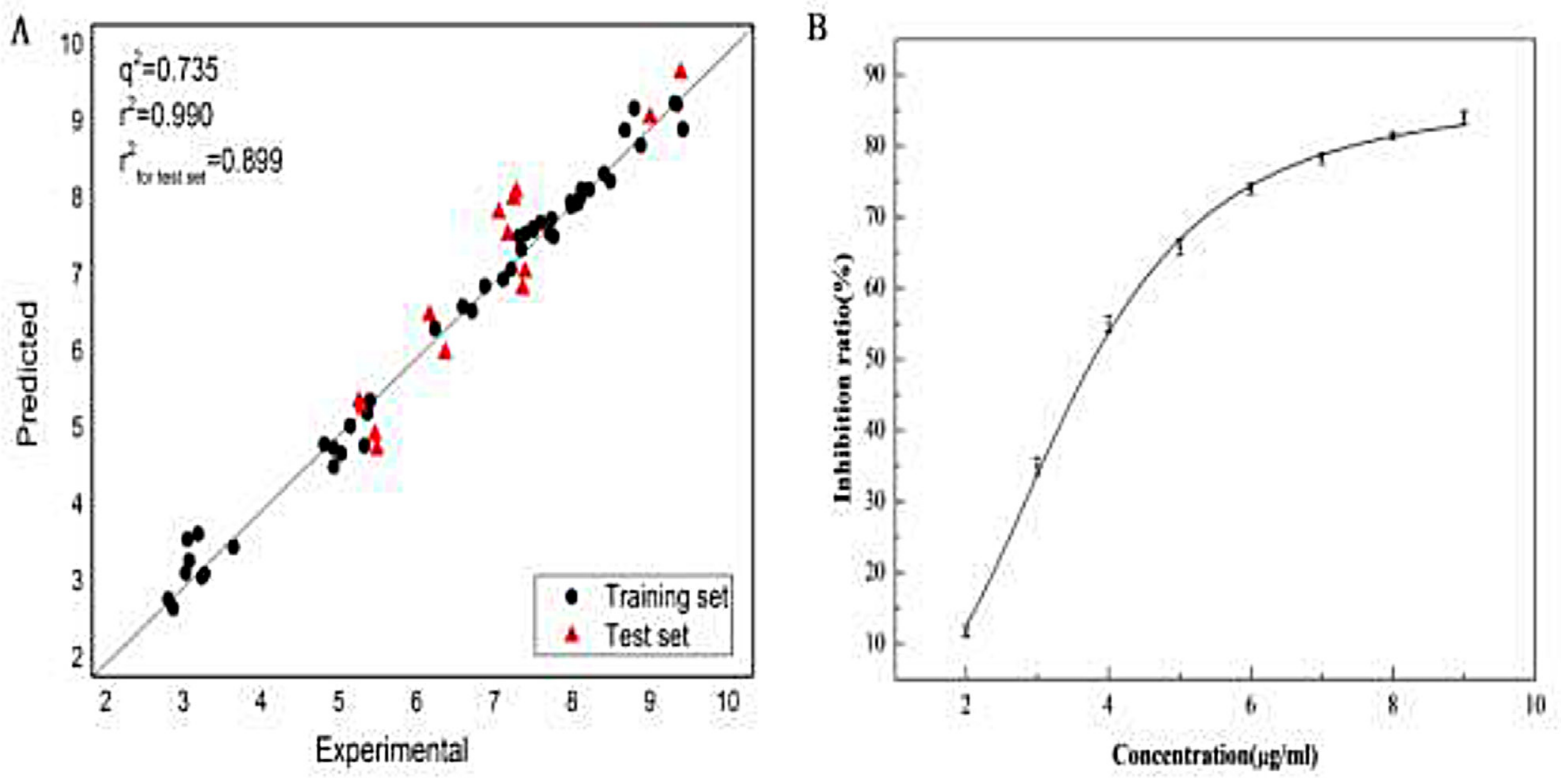
Plot (**A**) of the experimental data versus the predicted values andtheplot (**B**) of inhibition ratio at different concentrations of FB to the AChE.

Subsequently, the Ellman method was used to validate the predicted result. The inhibition ratio of FB in eight different concentrations (2.0, 3.0, 4.0, 5.0, 6.0, 7.0, 8.0 and 9.0 μg/mL) to AChE and the IC_50_ of FB were calculated and shown Figure 6. The IC_50_ of Huperzine A as a positive control to AChE was also measured. As an outcome, the half inhibitory concentration of FB and Huperzine A was obtained (IC_50FB_ = 3.478 ± 0.09μg/ml, IC_50huperzine_ = 74.8 ± 0.08 nmol/L), which is quite consistent with the reported result in literature (IC_50_ = 74.0 ± 0.06 nmol/L) [82]. The pIC_50_ of FB was obtained (pIC_50_= 5.22) by calculation, which was in the same order of magnitude with the predicted results.

### Clues for enhancing biological activity

**Figure 7:**
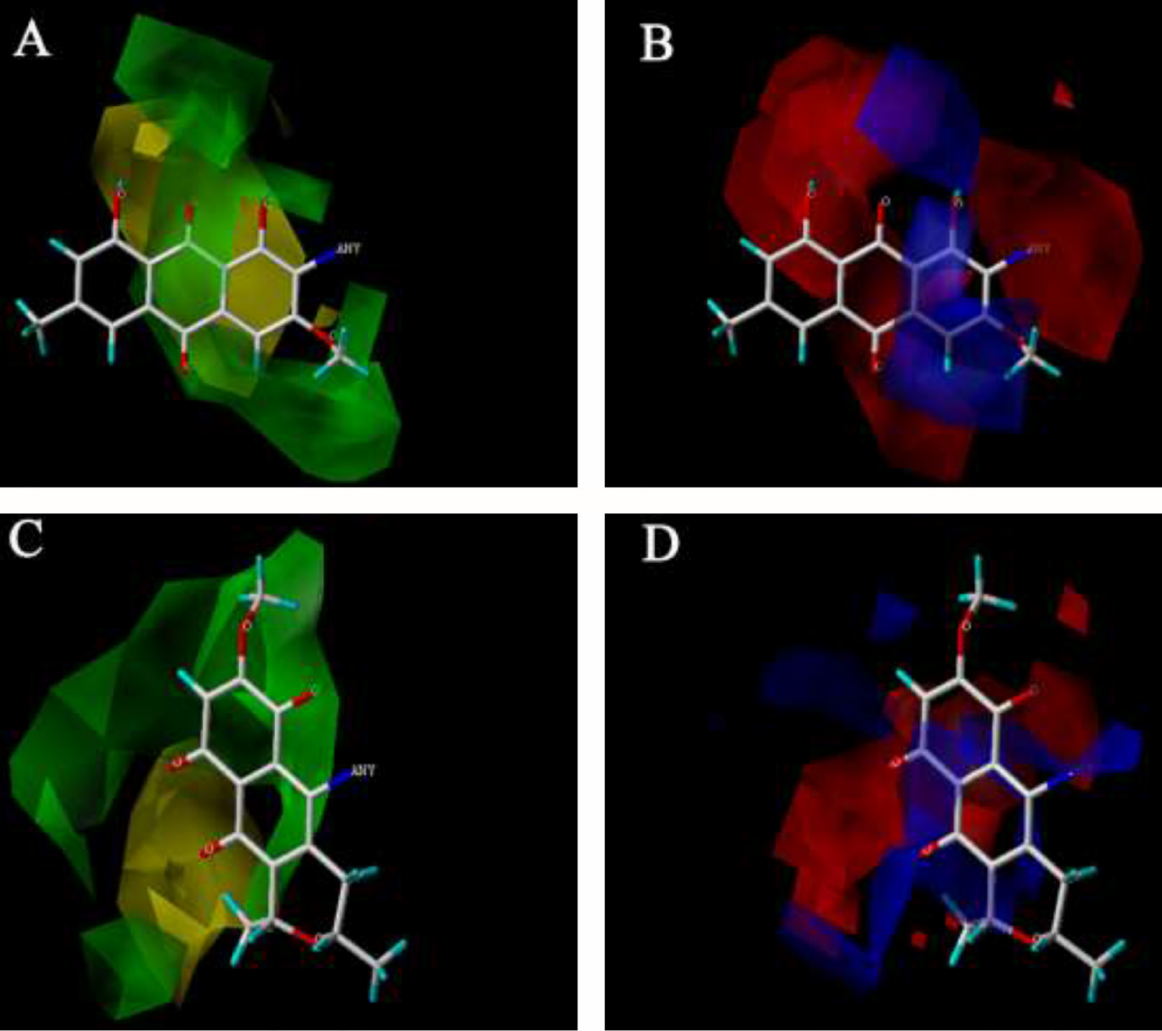
The 3D contour mapsof the TopomerCoMFAmodelfor R1 and R2 of FB The steric contour maps of R1 (**A**) and R2 (**C**); the electrostaticcontour maps of R1 (**B**) and R2 (**D**).

Based on the 3D contour maps obtained by the Topomer CoMFA model for R1 and R2 of FB, we can analyze how to further enhance the biological activity of FB. Shown in Figure 7 are the steric field contours (yellow and green), and the electrostatic field contours (red and blue). The former indicates that introducing large volume groups can improve the FB's activity, while the latter that adding the positive-charged groups can improve the FB's activity.

## MATERIALS AND METHODS

The FB samples (purity ≥ 98%) were purchased from the key chemical laboratory of natural products, Chinese Academy of Sciences. All other reagents were of analytical grade and the experimental water was double distilled water.

The fluorescence spectrum was obtained using F-7000 fluorescence spectrometer (Hitachi High-Technologies Corporation, Japan). An ultra micro-spectrophotometer was used to measure the OD260/OD280 absorbance ratio. The UV-vis absorption spectra were recorded on Cary100 UV-vis spectrophotometer (Varian, Australia). The circular dichroism spectra were measured on J-815 spectropolarimeter (Jasco, Japan). And an iMark microplate reader (BIO-RAD, America) was used to measure the inhibition ratio.

700 μL AChEsolution (pH = 7.4) was added to 1mL quartz cuvette. And 1μL FB solution (1 × 10^−5^M, the cumulative volume is less than 10μL) was dropped with micro-injector successively, and the solution was allowed to stand for 8 min to equilibrate. The emission spectra from 250 nm to 450 nm were recorded upon the excitation wavelength range of 230 nm and 280 nm at 298 K, 303 K and 308 K, respectively.

Three-dimensional fluorescence spectra of the AChE and the FB-AChE complexes (1:1, molar ratios) were obtained upon the excitation wavelength range of 200 nm-480 nm and the emission wavelength range of 220 nm-500 nm.

The ultraviolet absorption spectra of the AChE and FB before and after the reaction in the range of 200–800 nm were recorded. And 1μL FB solution (2 × 10^−4^ M) was dropped to 3 mL AChE solution (6.7 × 10^−8^ M) with micro-injector successively, and the solution was allowed to stand for 4min. In addition, the absorption spectra of FB (5 × 10^−8^ M) were recorded.

The CD spectra of AChE upon the far ultraviolet wavelength range of 190 nm–230 nm were measured at room temperature before and after the reaction with FB, respectively. The AChE solution (3.3 × 10^−8^ M) was added dropwise 2 × 10^−6^ M FB solution. Scanning speed was 50 nm/min, and the slit width was 2.0 nm. The measured data were the average of three measurements. In addition, the changes of the content of AChE in different secondary structure were quantitatively analyzed by means of the CONTINLL algorithms derived from CDPro software.

The protein structure of the AChE (PDB ID: 1QTI) was taken from the protein data bank (http://www.rcsb.org/pdb). The structure of FB was created using ChemDraw. The molecular docking was conducted by using the Tripos molecular modeling package in SYBYL-X-2.0.

The operation of TopomerCoMFA by combining Topomer technique and CoMFA technology can overcome the alignment problem of CoMFA [83]. In this work, a data set containing 61 inhibitors from [84–89] were collected, followed by studying the 3D QSAR of 46 kinds of compounds with the method of TopomerCoMFA by with SYBYL-X-2.0. A total of 61 compounds were randomly divided into a training dataset (47 compounds) and a testing dataset (14 compounds). The 3D QSAR model [90] was built to predict and analyze the biological activity of FB.

The biological activity of FB was measured with the Ellman method according to the following steps.

Sample group: 140 μL PBS buffer solution (0.1M, pH = 8.0), 20 μL sample solution and 20 μL AChE solution (0.28 U/mL) were introduced into the enzyme label plate in turn. The solution was preserved at 4°C for 20 min after mixing. Then 10 μL dithiobis nitrobenzoic acid (DTNB, 7.5 mM) and 10 μL acetylthiocholine (ATCI, 10 mM) were added. After 20 minutes, the absorbance was measured at λ = 405 nm.

Sample-out group: 20 μL enzyme solution was replaced by 20 μL PBS buffer solution (pH = 8.0), ceteris paribus.

Blank group: 20 μL sample solution was replaced by 20 μL PBS buffer solution (pH = 8.0), ceteris paribus.

Completely inhibit groups: substituting 20 μL of the sample solution with 20 μL huperzine solution (100 μg/mL, anhydrous methanol solution to 1 mg/mL as mother liquor, then were diluted using PBS (pH = 8.0)), ceteris paribus.

And the inhibition ratio can be calculated according to the equation below

$$\text{R(\%)=}\frac{\left(A_{3}-A_{4})-(A_{1}-A_{2}\right)}{\left(A_{3}-A_{4}\right)}$$ (4)

where R is the inhibition ratio, *A*_1_ the absorbance of sample group, *A*_2_ the absorbance of sample-out group, *A*_3_ the absorbance of blank group, and *A*_4_ the absorbance of completely inhibit group.

## CONCLUSIONS AND PERSPECTIVE

It is known that several efficient AChEIs have been extracted from medicinal plants. In comparison with the synthetic drugs, those extracted from plants have the following remarkable advantages: stronger activity, higher selectivity, and lower toxicity. Consequently, it is currently a very hot topic and main trend to search for the efficient AChEI from plants.

In this study, the interaction between FB and AChE was investigated. And the fluorescence, UV-vis, and CD spectrum were applied to study the interaction between FB and AChE under the physiological conditions (pH = 7.4). The results clearly demonstrated that the fluorescence quenching of AChE by FB was static quenching. Moreover, there was about one binding site and the distance between the FB and AChE was calculated (r = 3.502 nm). The results of CD and three-dimensional fluorescence indicated that FB could change the microenvironment of AChE. Furthermore, the results of molecular docking showed the binding site and the combining capacity were fully consistent with the experimental observations. It can be concluded from these competing facts that FB can be considered as a potential AChEI.

This study has also provided some useful clues and hints for designing more effective drugs against Alzheimer's disease through the Topomer CoMFA model such as the big size and positive-charged groups.

As demonstrated in a series of recent publications (see, e.g., [91–107]) in reporting new findings or approaches, user-friendly and publicly accessible web-servers will significantly enhance their impacts [55]; otherwise, their usage would be quite limited [108]. We shall make efforts in our future work to provide a web-server to display the findings reported in this study.”

## SUPPLEMENTARY MATERIALS FIGURES AND TABLES


