# Supplementary material for: Small molecular floribundiquinone B derived from medicinal plants inhibits acetylcholinesterase activity
Source: Oncotarget. 2017 Jul 11;8(34):57149–62. doi: 10.18632/oncotarget.19169 (PMC5593632; doi:10.18632/oncotarget.19169)
Supplement: Supplementary file 1 [file oncotarget-08-57149-s001.pdf]

Small molecular floribundiquinone B derived from medicinal plants inhibits acetylcholinesterase activity

SUPPLEMENTARY MATERIALS

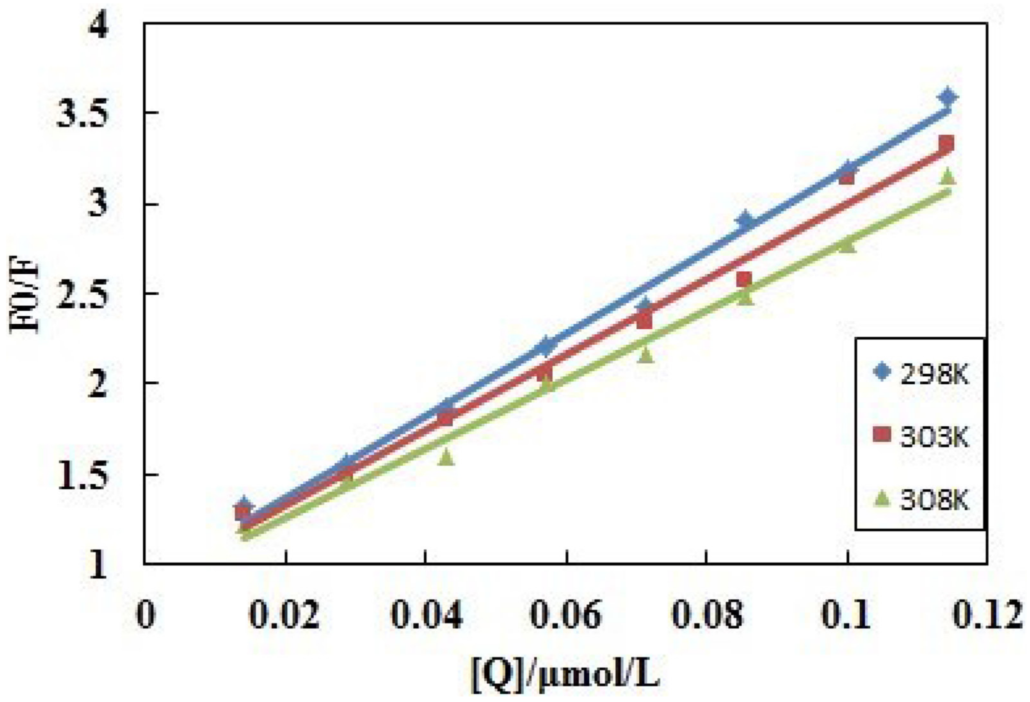

Supplementary Figure 1: The unmodified Stern–VolmercurvesofAChE fluorescence quenched by FB.

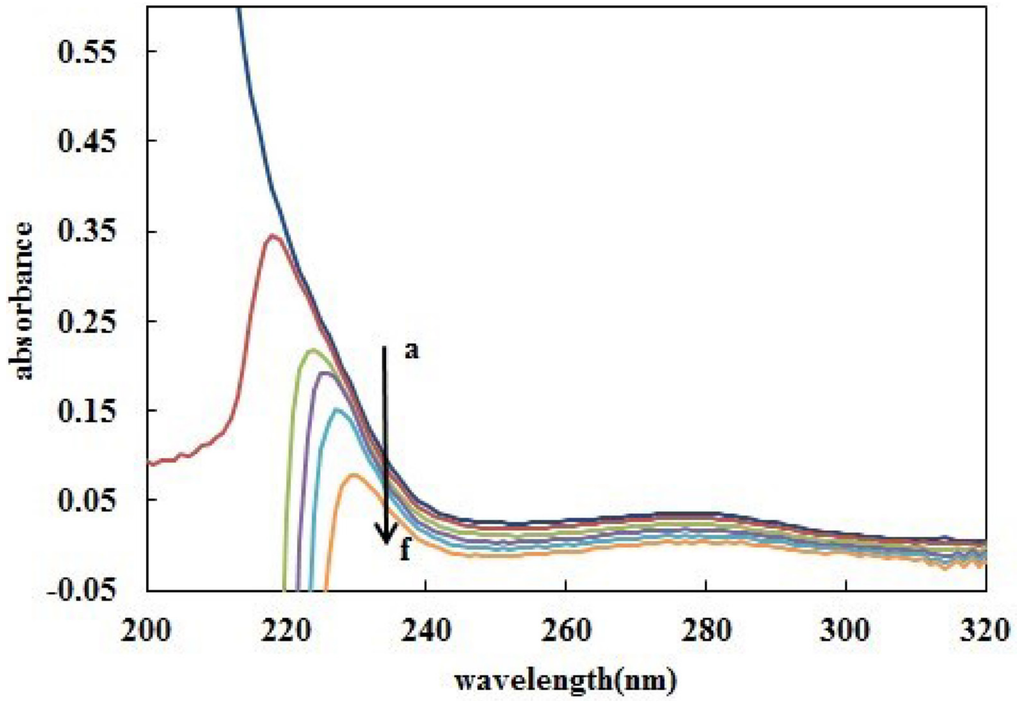

Supplementary Figure 2: The UV–vis absorption spectra of AChE in the presence of different FB concentration. $C_{AChE,a}=6.7 \times 10^{-8}$ M; $C_{FB,a\rightarrow f}$: 0, 0.67, 1.33, 2.67, 3.33, and 4×10^{-7} M; T = 303 K.

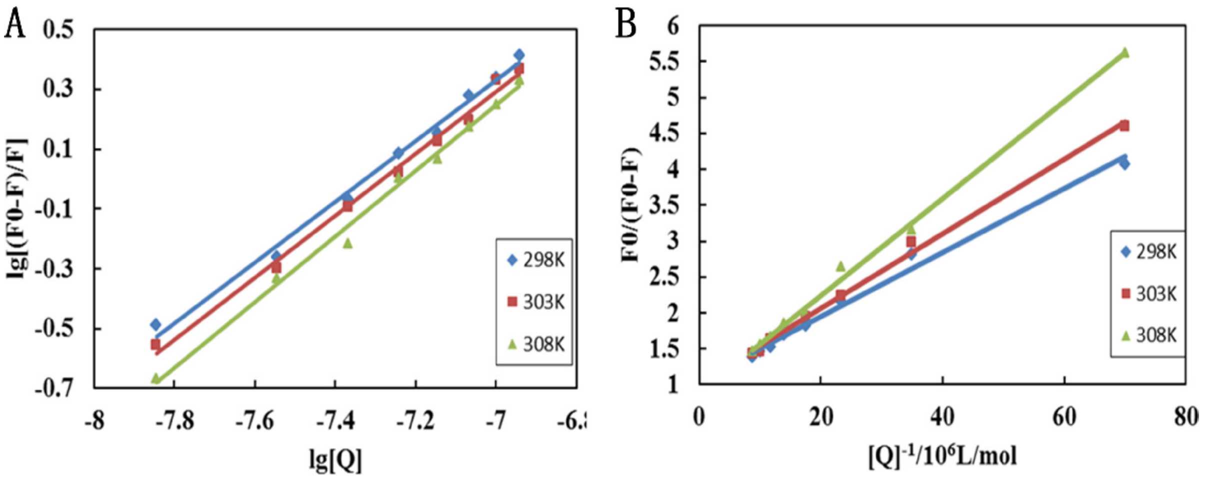

Supplementary Figure 3: (**A**) The double logarithmic curves for the AChE fluorescence quenched by FB, and (**B**) their modified Stern–Volmer curves.

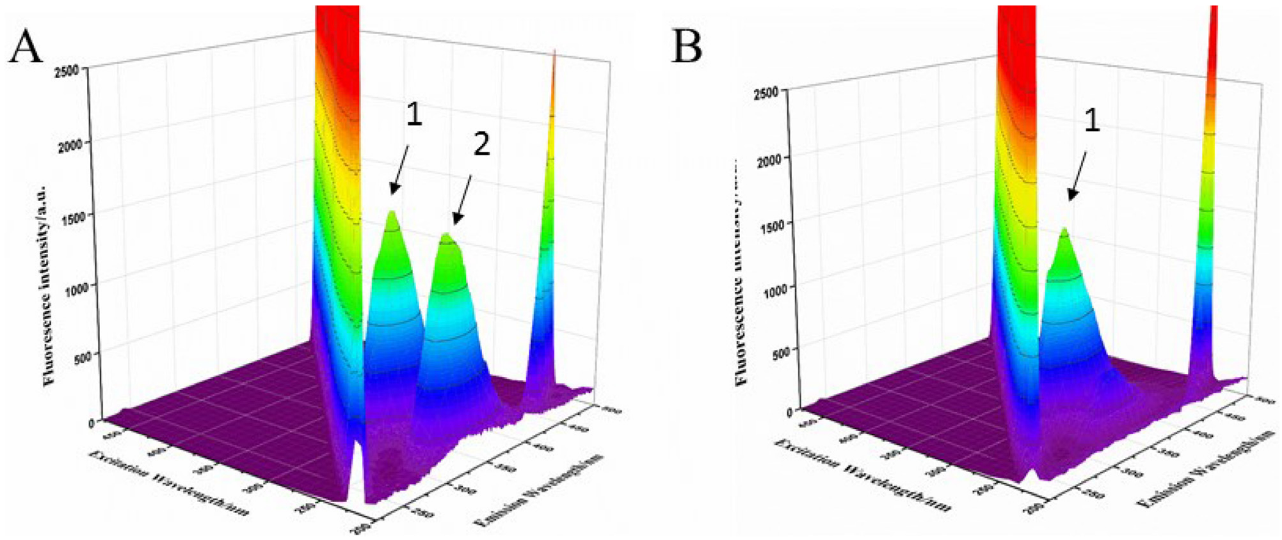

Supplementary Figure 4: The 3-dimensional fluorescence spectra for (**A**) the AChE system, and (**B**) the FB-AChE system.
